# microT-CNN: an avant-garde deep convolutional neural network unravels functional miRNA targets beyond canonical sites

**DOI:** 10.1093/bib/bbae678

**Published:** 2024-12-31

**Authors:** Elissavet Zacharopoulou, Maria D Paraskevopoulou, Spyros Tastsoglou, Athanasios Alexiou, Anna Karavangeli, Vasilis Pierros, Stefanos Digenis, Galatea Mavromati, Artemis G Hatzigeorgiou, Dimitra Karagkouni

**Affiliations:** Department of Computer Science and Biomedical Informatics, University of Thessaly, Papasiopoulou 2-4, Lamia 35131, Greece; Hellenic Pasteur Institute, 127 Vasilissis Sofias Avenue, Athens 11521, Greece; DIANA-Lab, Department of Computer Science and Biomedical Informatics, University of Thessaly, Papasiopoulou 2-4, Lamia 35131, Greece; DIANA-Lab, Department of Computer Science and Biomedical Informatics, University of Thessaly, Papasiopoulou 2-4, Lamia 35131, Greece; Hellenic Pasteur Institute, 127 Vasilissis Sofias Avenue, Athens 11521, Greece; DIANA-Lab, Department of Computer Science and Biomedical Informatics, University of Thessaly, Papasiopoulou 2-4, Lamia 35131, Greece; Department of Computer Science and Biomedical Informatics, University of Thessaly, Papasiopoulou 2-4, Lamia 35131, Greece; Hellenic Pasteur Institute, 127 Vasilissis Sofias Avenue, Athens 11521, Greece; DIANA-Lab, Department of Computer Science and Biomedical Informatics, University of Thessaly, Papasiopoulou 2-4, Lamia 35131, Greece; DIANA-Lab, Department of Computer Science and Biomedical Informatics, University of Thessaly, Papasiopoulou 2-4, Lamia 35131, Greece; DIANA-Lab, Department of Computer Science and Biomedical Informatics, University of Thessaly, Papasiopoulou 2-4, Lamia 35131, Greece; DIANA-Lab, Department of Computer Science and Biomedical Informatics, University of Thessaly, Papasiopoulou 2-4, Lamia 35131, Greece; DIANA-Lab, Department of Computer Science and Biomedical Informatics, University of Thessaly, Papasiopoulou 2-4, Lamia 35131, Greece; Department of Computer Science and Biomedical Informatics, University of Thessaly, Papasiopoulou 2-4, Lamia 35131, Greece; Hellenic Pasteur Institute, 127 Vasilissis Sofias Avenue, Athens 11521, Greece; DIANA-Lab, Department of Computer Science and Biomedical Informatics, University of Thessaly, Papasiopoulou 2-4, Lamia 35131, Greece; Department of Pathology, Beth Israel Deaconess Medical Center, 330 Brookline Ave, Boston, MA 02215, United States; Harvard Medical School, 229 Longwood Ave, Boston, MA 02115, United States; Broad Institute of MIT and Harvard, 415 Main St, Cambridge, MA 02142, United States

**Keywords:** microRNA, CNN, deep learning, target prediction, CLIP-seq, CLASH, viral-encoded miRNAs, EBV, KSHV

## Abstract

microRNAs (miRNAs) are central post-transcriptional gene expression regulators in healthy and diseased states. Despite decades of effort, deciphering miRNA targets remains challenging, leading to an incomplete miRNA interactome and partially elucidated miRNA functions. Here, we introduce microT-CNN, an avant-garde deep convolutional neural network model that moves the needle by integrating hundreds of tissue-matched (in-)direct experiments from 26 distinct cell types, corresponding to a unique training and evaluation set of >60 000 miRNA binding events and ~30 000 unique miRNA–gene target pairs. The multilayer sequence-based design enables the prediction of both host and virus-encoded miRNA interactions, providing for the first time up to 67% of direct genuine Epstein–Barr virus– and Kaposi’s sarcoma–associated herpesvirus–derived miRNA–target pairs corresponding to one out of four binding events of virus-encoded miRNAs. microT-CNN fills the existing gap of the miRNA–target prediction by providing functional targets beyond the canonical sites, including 3′ compensatory miRNA pairings, prompting 1.4-fold more validated miRNA binding events compared to other implementations and shedding light on previously unexplored facets of the miRNA interactome.

## Introduction

MicroRNAs (miRNAs) serve as central regulators of gene expression, associated with diverse cellular processes from growth and differentiation to disease progression [1]. Upon loading into Argonaute (AGO) proteins, miRNAs orchestrate post-transcriptional gene regulation through target cleavage, degradation, or translational suppression [2]. Significant effort has been made over the past few decades to decipher the role of virally encoded miRNAs, their interactions with the host target, and their role in manipulating host gene expression to favor viral replication and the evasion of immune response [2, 3]. Importantly, given the miRNAs’ broad potential in diagnostic, prognostic, and therapeutic applications, deciphering their targets is paramount [3].

Over the past two decades, considerable efforts have been directed toward developing computational tools for predicting miRNA targets. Existing implementations take into account distinct characteristics [4, 5], including miRNA-seed (positions 2–8) pairing with full or partial target complementarity, employing diverse statistical and machine learning approaches, and trained/evaluated on diverse experimental data [6], including (in)direct specific or high-throughput techniques [6, 7]. The advent of high-throughput direct experimental techniques, such as AGO-CLIP-seq (Argonaute cross-linking and immunoprecipitation sequencing) and CLASH (Crosslinking, Ligation, And Sequencing of Hybrids), in the past decade has facilitated the thorough characterization of numerous AGO-binding regions. These advancements have illuminated functional non-canonical seed-based, as well as 3′ compensatory miRNA-binding pairings within both the 3′ untranslated region (UTR) and coding regions (CDS), serving as invaluable resources for deploying miRNA target prediction models [6–8].

Targetscan [9], trained on 74 microarray datasets, is considered one of the state-of-the-art models and predicts canonical 3′ UTR miRNA target sites with a cumulative context and/or an aggregated conservation score. On the other end, DIANA–microT-CDS [10] predicts miRNA–target pairs on CDS and 3′ UTR regions, while it was the first model to utilize both miRNA perturbation and AGO-PAR-CLIP (Photoactivatable-Ribonucleoside-Enhanced Crosslinking and Immunoprecipitation) experiments to capture the functional potential of direct miRNA binding events. PACCMIT [11] and PACCMIT-CDS [12] are more recent algorithms. Based on an overrepresentation ranking system, they can predict seed-based miRNA target sites in the 3′ UTR and CDS regions, respectively. On the other end, MBSTAR [13] incorporates >40 characteristics, including sequence, structural, and energy features, and mirMark [14] integrates an extensive list of site and UTR-relevant features. Both models were trained and evaluated on experimentally supported miRNA–gene targets derived from public repositories [15–18]. More recent prediction methods integrate deep learning approaches, such as miRAW [19], cnnMirTarget [20], and TargetNet [19]. Many of the aforementioned models have primarily been trained using miRNA perturbation experiments, which may under-represent the direct effect of predicted miRNA binding sites [6]. Few of them, including miRAW [20], cnnMirTarget [21], and DIANA-microT-CDS [22], have been trained only on a small number of AGO-CLIP-Seq experiments derived from specific cell types and tissues, thereby not comprehensively capturing the miRNA interactions [6, 23]. Most methods disregard miRNA binding sites with imperfect complementarity or those residing within coding regions to mitigate low performance, consequently covering a limited number of genuine miRNA binding events [6, 23]. Notably, there is a lack of methods identifying interactions of virus-encoded miRNAs, primarily due to the only recent establishment of high-throughput direct techniques [7, 24].

**Figure 1:**
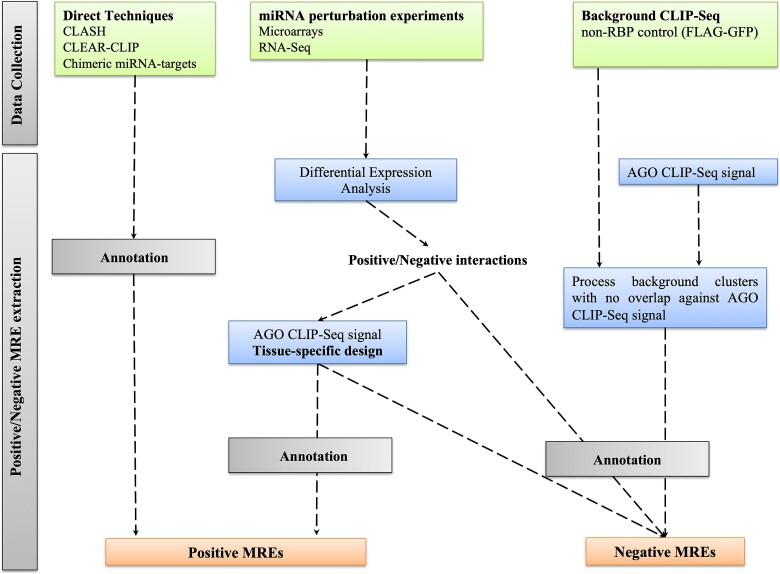
Overview of miRNA-target positive/negative binding sites identified by different indirect/direct, low, and high-throughput experiments. miRNA-targeted regions derived from miRNA perturbation datasets presented an overlap with AGO-bound enriched regions from at least one AGO-CLIP-seq library. Datasets have been combined under tissue-specific rules. No overlap between positive and negative miRNA–gene interactions and their related miRNA binding sites was allowed.

**Figure 2:**
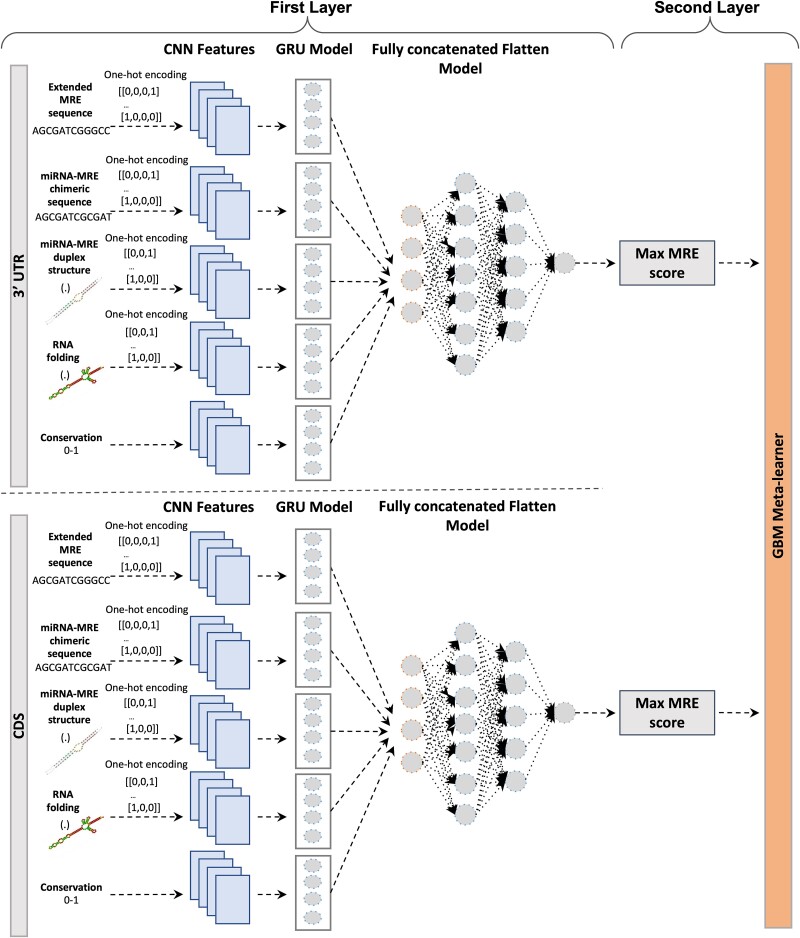
Overview of the microT-CNN framework. The framework follows a two-layer sophisticated approach. The first layer integrates two distinct models to predict miRNA binding sites on (top) 3′ UTR and (bottom) CDS regions separately. Each model is composed of five distinct “expert” branches, each designed to model different aspects of the input data: (a) 150 nt-long MRE-extended nucleotide sequences, (b) 53 nt-long miRNA-MRE chimeric sequences that capture the combined nucleotide sequence of the miRNA (23nts) and its target MRE (30nts), (c) miRNA-MRE duplex structure hybrids of length 60 that reflect the duplex structure in a dot-bracket notation formed by miRNA binding to its target, (d) RNA folding regions of 150 length in a dot-bracket notation that provide information on the secondary structural context and accessibility of the MRE region, and (e) phastCons conservation scores (range of 0–1) for the extended 150 nt-long MRE region. For feature branches a, b, c, and d, one-hot encoding was applied to convert nucleotide and dot-bracket sequences into a binary format compatible with the CNN-GRU model. The extracted features from the convolutional branches were utilized as an input in GRU classifiers and flattened, concatenated, and forwarded to the dense part of the architecture that produces the final prediction scores for the MREs. In the second layer, the top-scored MREs on 3′ UTR and CDS regions were aggregated in a GBM meta-learner to score the miRNA–gene interactions.

Here, we introduce microT-CNN, a next-generation DCNN (Deep Convolutional Neural Network) framework that fills the existing gap of the multifaceted miRNA-targeting problem by providing functional (non-)canonical seed-based and 3′ compensatory miRNA binding events, leveraging the high-yield AGO-CLIP tissue-specific miRNA-binding accuracy, combined with the miRNA–target functionality from gene expression experiments (Fig. 1). The model encompasses a multilayered structure, mapping the exact miRNA binding location and the functional potential of the miRNA gene targets (Fig. 2). The first layer encompasses a sophisticated approach that integrates five expert “agents” that serve as independent modes, each following a CNN–gated recurrent unit (CNN-GRU) architecture (Fig. 2). The five independent modes learn miRNA binding patterns directly from (i) the raw extended MRE (miRNA Recognition Element) sequences, (ii) the combined chimeric miRNA and MRE sequences, and (iii) the miRNA-binding secondary structure hybrids, as well as the RNA-accessibility and conservation footprints of the extended miRNA binding region. The layer was trained on a unique set of miRNA binding events by integrating >60 000 miRNA binding sites derived from the analysis of ~130 AGO-CLIP-seq experiments combined with ~70 tissue-matched miRNA perturbation experiments. The ensemble multi-agent approach facilitates the extraction of informative, hidden patterns separately in 3′ UTR and CDS regions and provides miRNA binding sites beyond the miRNA seed region, including 3′ compensatory miRNA pairings, previously disregarded, capturing the whole spectrum of interactions. MREs from 3′ UTR and CDS regions are aggregated in a gradient-boosted model (GBM) meta-learner, trained on ~4000 positive and negative miRNA interactions derived from miRNA transfection experiments to provide functional targets (Fig. 2).

microT-CNN is the first model to be evaluated on ~3000 (q)CLASH-derived (quick crosslinking, ligation, and sequencing of hybrids) virally encoded miRNA interactions and to achieve high performance by accurately predicting ~50% of genuine Epstein–Barr virus (EBV) and Kaposi’s sarcoma–associated herpesvirus (KSHV) miRNA targets, and up to 67% of miRNA binding sites corresponding to one out of four virally encoded MREs.

microT-CNN is a next-generation *in silico* model providing >70% direct miRNA targets on a comprehensive test set of genuine MREs. Our model detects 1.4-fold more validated miRNA binding sites than leading implementations, marking a significant breakthrough in miRNA–target annotation and unveiling previously unexplored patterns of the miRNA interactome.

To ensure compatibility and stability, microT-CNN is packaged as a Docker image and can be accessed on GitHub [https://github.com/dianalabgr/microT-CNN.git].

## Materials and Methods

### Dataset collection

microT-CNN was trained and tested against an extensive set of miRNA binding sites (Supplementary Tables 1–5) retrieved from the analysis of low-/high-throughput techniques across 46 distinct cell types and tissues (Fig. 1). Specifically, miRNA-targeted regions were extracted by combining AGO-CLIP-seq libraries with tissue-matched miRNA perturbation gene expression data after specific miRNA transfection, silencing, or knockout (Supplementary Table 1). AGO-enriched regions derived from AGO-CLIP-seq data, 57 HITS-CLIP (High-Throughput Sequencing of RNA isolated by CrossLinking Immunoprecipitation), and 81 PAR-CLIP (Photoactivatable-Ribonucleoside-Enhanced Crosslinking and Immunoprecipitation) libraries were coupled with differentially expressed mRNAs extracted from 49 miRNA-specific high-throughput experiments (42 microarrays, 31 RNA-seq) across 26 different cell types and 10 tissues (Supplementary Tables 1 and 6–8). This process enabled the formation of 8497 positive and 2305 negative MREs (Supplementary Tables 2 and 3). Two published non-RBP (FLAG-GFP) background PAR-CLIP libraries, previously described [25], were incorporated into our pipeline to retrieve 8682 non-AGO-bound miRNA binding sites. Potential MREs residing in negatively correlated miRNA–gene interactions from miRNA perturbation experiments, non-overlapping with AGO-enriched regions from CLIP-seq experiments, complemented the set of negative MREs (Supplementary Tables 2 and 3). A high-quality set of 14 366 direct miRNA-MRE chimera fragments from CLASH and CLEAR-CLIP (Covalent Ligation of Endogenous Argonaute-bound RNAs–Cross-Linking and Immunoprecipitation) experiments, indexed in DIANA-TarBase v8.0 [26] and DIANA-LncBase v3.0 [27] was utilized for the training and evaluation of the model (Supplementary Tables 2 and 3). The retrieved miRNA binding sites were annotated against a reference set of coding and 3′ UTR exons. In cases of multiple transcript–gene associations, principal isoforms were selected according to APPRIS criteria [28] and the transcript with the longest 3′ UTR. An independent dataset of 2113 positive and 2009 negative miRNA–gene interactions, derived from miRNA transfection RNA-seq experiments, was utilized to train the meta-learner and the mRNA–target prediction (Supplementary Table 4).

### Analysis of high-throughput experiments

High-throughput AGO-CLIP-seq libraries and miRNA perturbation experiments were analyzed following best practices [27]. In brief, raw AGO-CLIP-seq and RNA-seq libraries were quality-checked, preprocessed, and aligned against the reference human genome, GRCh38 [29], as previously described [25]. For the model training, the microCLIP framework [25] was utilized to detect AGO-enriched regions, and only those with microCLIP score >0.5 were retained. A modified parallelized version of the BBMap aligner [30] was utilized to retrieve potential MREs in the AGO-CLIP-seq peaks.

RNA quantification was conducted at the transcript level, using Salmon quasi-mapping mode [31], followed by differential expression with Sleuth [31, 32]. Microarray-analyzed datasets were obtained from DIANA-LncBase v3.0 [27]. For both RNA-seq and microarray miRNA perturbation experiments, a threshold of 1.5-fold change (FDR < 0.05 where applicable) was used to retrieve positive and negative miRNA targets. The retrieved MREs from the AGO-enriched regions were combined with the aforementioned miRNA transfection/knockout positive and negative targets in a tissue-specific way to obtain positive and negative MREs for the model training (Supplementary Table 1).

### (q)CLASH interactions of virally encoded microRNAs

Interactions between virally encoded miRNAs and host transcripts experimentally supported via (q)CLASH experiments were extracted from DIANA-TarBase v9.0 [7]. Briefly, (q)CLASH methods directly assess miRNA binding via a ligation step to join each interacting miRNA and respective targeted RNA into chimeric fragments, which are then subjected to next-generation sequencing. In TarBase 9.0, Hyb workflow [33] and RNAup [34] were used to analyze (q)CLASH libraries *de novo* against tailored databases containing host miRNA/transcript sequences as well as the virally encoded miRNA sequences. MREs exhibiting a negative minimum free energy (<0 kcal/mol) and supported by more than one chimeric read were retained.

### microT-CNN framework

#### Description of the algorithm

microT-CNN framework follows a two-layer approach and can accurately characterize miRNA interactions at the MRE and gene levels (Fig. 2). Users provide the miRNA sequences, the gene sequences, and conservation scores in a bigWig format, and the model predicts the miRNA–gene interactions and their exact miRNA-binding location on 3′ UTR and CDS transcript regions.

microT-CNN operates on the raw transcript and miRNA sequences. The algorithm extracts (i) 150 nt-long raw MRE sequences (30 nt-long MREs and 60 nt-long upflank/downflank regions), (ii) 53 nt-long miRNA-MRE chimeric sequences (30 nt-long MRE sequences, 23 nt-long miRNA sequences), (iii) miRNA–target duplex structure hybrids with a length of 60 in a dot-bracket notation, (iv) RNA folding of the 150 nt-long MRE sequences in a dot-bracket notation, and (v) the conservation phastCons scores of the extended 150 nt-long MRE regions. A sophisticated two-layer approach utilizes the extracted regions: (i) a CNN architecture combined with GRU to extract informative patterns hidden in the raw regions and to accurately score the putative miRNA binding sites and (ii) a meta-learner that combines the retrieved MRE scores from the 3′ UTR and CDS regions to characterize high-quality miRNA–gene interactions.

The pipeline initially utilizes a modified, multithreaded version of the BBmap aligner [30] to scan the transcript sequences for putative miRNA binding sites. An extensive set of (non-)canonical binding sites is supported, including 6mer- to 9mer, centered, and 3′ compensatory sites, to capture the exact range of miRNA binding regions (Supplementary Table 5). miRNA–target hybrids of the putative sites and the RNA folding of the MRE region, extended to 60nts downflank and upflank regions, were calculated with RNAduplex and RNAfold, respectively, from the Vienna package [35]. phastCons precomputed scores from genome-wide multiple alignments were downloaded from the UCSC (University of California Santa Cruz) Genome Browser repository [36] in bigWig format and were utilized to deduce respective evolutionary rates. Conservation signals were retrieved from miRNA-targeted sites and their flanking regions (60nts upflank and downflank).

#### MRE level

The first layer integrates a hybrid CNN-GRU architecture built with TensorFlow and Keras [37]. The model was trained and evaluated separately on CDS and 3′ UTR regions using a high-quality set of 67 379 MREs (36 405 3′ UTR, 30 974 CDS, Supplementary Table 3). The architecture includes five distinct “expert” branches, each designed to model different aspects of the input data: (i) 150 nt-long MRE-extended sequences that represent extended nucleotide sequences (60nts downflank and upflank) surrounding the 30nts MREs, (ii) 53 nt-long miRNA-MRE chimeric sequences that capture the combined nucleotide sequence of the miRNA (23nts) and its target MRE (30nts), (iii) miRNA-MRE duplex hybrids with a length of 60 that reflect the duplex structure in a dot-bracket notation formed by miRNA binding to its target, (iv) 150 nt-long RNA folding regions (extended MRE regions, 60nts downflank and upflank) in a dot-bracket notation that provide information on the secondary structural context of the RNA, and (v) phastCons conservation scores (range [0–1]) for the extended 150 nt-long MRE region. For the raw sequence data (branches a, b, c, and d), one-hot encoding was applied to convert nucleotide and dot-bracket sequences into a binary format compatible with the CNN-GRU model.

The normalized extracted features from the convolutional branches were utilized as inputs for GRU layers, which capture sequential dependencies. The outputs from the GRU layers were then flattened, concatenated, and passed to the dense layers, which produced the final prediction scores for the MREs. Regularization techniques, including dropout and batch normalization, were employed at each layer to improve generalization. A grid search was performed to optimize hyperparameters, such as learning rate, dropout rate, the number of filters in the convolutional layers, and the number of GRU units.

#### Convolutional-Gated Recurrent Unit branches

The model uses distinct specialized convolutional branches to extract meaningful biological information from different types of input sequences. Each branch is configured to maximize the model’s ability to capture relevant features: (i) the MRE branch, consisting of two convolutional layers with filter sizes of 35 and 50 and strides of 2 and 3; (ii) the miRNA-MRE chimeric branch, using three convolutional layers with filter sizes of 16, 32, and 62 and strides of 2, 3, and 4; (iii) the miRNA-MRE duplex structure hybrid branch, containing three convolutional layers with filter sizes of 16, 32, and 62 and strides of 2, 3, and 4; (iv) the RNA folding branch, using two convolutional layers with filter sizes of 35 and 50 and strides of 4 and 5; and (v) the conservation branch, which includes three convolutional layers with filter sizes of 20, 40, and 60 and strides of 3, 4, and 5. All branches use max-pooling with a pool size of 2 to focus on the most important features without applying padding, which ensures the extraction of contiguous patterns in the sequences.

After feature extraction through the convolutional layers, the outputs are fed into GRU layers with 24 units. The GRU layers help capture sequential relationships in the data, and a dropout rate of 0.1 is applied to prevent overfitting.

#### Flattened model

The outputs from all the CNN-GRU branches are combined into a single vector by flattening and concatenating them. This vector is then passed to the dense network, which consists of three layers with 90, 55, and 35 nodes, respectively. A dropout rate of 0.1 is applied for regularization. The dense layers use Leaky ReLU activation functions, and the final output layer uses a sigmoid activation to produce the prediction probability for MREs. The model is trained over 200 epochs, with early stopping enabled (patience of 15 epochs) and a learning rate of 10^−4^.

#### Metaclassifier—Gene level

An independent dataset consisting of 2113 positive and 2009 negative miRNA–gene interactions, derived from RNA-seq miRNA transfection experiments, was used to train microT-CNN at the gene level. The putative MREs and features from the first layer were calculated using the core microT-CNN algorithm to generate MRE scores for all possible miRNA binding sites. The maximum MRE score per interaction was calculated separately for the 3′ UTR and CDS regions in both the positive and negative sets, and these scores were used as features to train a GBM meta-learner (Fig. 2). MREs with a score >0.5 for the first layer were retained for the positive set, while those with a score <0.5 were retained for the negative set. Five-fold cross-validation was applied to the training data to evaluate model accuracy and finalize the learning architecture. The microT-CNN training and the required computations for model optimization were carried out using multithreading.

#### Docker image

A microT-CNN Docker image was generated, leveraging Docker version 24.0.7 [38] to ensure compatibility and stability. Adhering to stringent specifications, precise version control of all packages was maintained to ensure consistency and reproducibility in miRNA target prediction [https://hub.docker.com/repository/docker/penny0lane/microt_cnn/general]. Our user-friendly approach provides clear documentation, and the accompanying code is accessible on GitHub [https://github.com/dianalabgr/microT-CNN.git].

### microRNA interactions from *de novo* target prediction models

To evaluate microT-CNN alongside other prediction algorithms (MBSTAR [13], miRAW [20], Targetscan v7 [9], cnnMirTarget [21], PACCMIT-CDS [12], microT-CDS [10], mirMark [14], and TargetNet [19]), we utilized a subset of chimeric fragments, AGO-PAR-CLIP, and microarray datasets from our test set (Supplementary Tables 2, 3, and6–8). The models were evaluated using the corresponding sets of miRNAs and genes. Precalculated miRNA–target interactions were utilized for Targetscan v7 and PACCMIT-CDS. Targetscan provided a Context++ score, where lower values indicate stronger miRNA–target interactions. PACCMIT-CDS provided canonical gene-level miRNA targets within 3′ UTR and CDS regions. We utilized two modes of the PACMIT-CDS model: one incorporating conservation scores and the other using binding energy scores. The respective miRNA binding site coordinates were converted to assembly hg38 and annotated to Ensembl v100 [39] and mirBase v22 [40].

For the remaining models, the input data were structured and run following developers’ instructions. The retrieved (non-)canonical MBSTAR miRNA binding sites resided solely on the 3′ UTR region, with a probability score of > 0.5. For mirMark, we executed the Docker image and retrieved 3′ UTR (non-)canonical miRNA binding sites with a probability score set at 0.5. TargetNet was executed using the publicly available code on GitHub, with the required conda environment installed as per the developers’ instructions. The model provided gene-level miRNA target prediction probability scores ranging from 0 to 1, with a cutoff threshold set at 0.5. miRAW provided scores ranging from −1 to 1 for canonical and non-canonical miRNA binding site predictions in the 3′ UTR region, while cnnMirTarget reported binding sites with scores exceeding 0.5. microT-CDS provided canonical and non-canonical binding site predictions, along with gene-level probability scores for both the 3′ UTR and CDS regions, with a probability cutoff score set at 0.5. To evaluate the models’ performance at the gene level, we aggregated the predicted MRE scores (sum of MRE scores) in cases where only miRNA binding site scores were available, specifically for MBSTAR, mirMark, miRAW, and Targetscan.

## Results

### A deep-convolutional approach for *de novo* microRNA target identification

microT-CNN is a next-generation model that accurately predicts (non-)canonical miRNA binding events beyond the seed miRNA region, including 3′ compensatory miRNA pairing events, as well as functional miRNA targets in 3′ UTR and CDS regions. The model was deployed by (i) integrating an extensive set of >60 000 miRNA binding sites and ~30 000 tissue-matched unique functional miRNA–gene interactions and (ii) adopting a multilayer sophisticated approach under a DCNN scheme (Figs 1 and 2).

microT-CNN model performance was initially evaluated in an independent set of 830 miRNA binding sites (415 positive miRNA-chimeric fragments and 415 negative miRNA binding sites), corresponding to 131 miRNAs and 727 unique genes (Supplementary Tables 2 and 3). The model demonstrated high specificity, sensitivity, precision, and recall for both miRNA–target (Specificity: 0.86, Sensitivity: 0.8, Precision: 0.817, Recall: 0.863) and MRE (Specificity: 0.86, Sensitivity: 0.85, Precision: 0.856, Recall: 0.863) detection. These metrics were achieved by applying optimal thresholds to the microT-CNN scores based on the Youden index (MRE prediction: 0.878, miRNA–target prediction: 0.724).

The model’s overall performance was robust, as reflected by the area under the curve (AUC) of 0.895 (95% CI: 0.874–0.916) for miRNA–target prediction and 0.922 (95% CI: 0.9–0.94) for MRE detection (Supplementary Fig. 1). Additional metrics demonstrated consistently high performance, with accuracy, F1 score, and area under the precision–recall curve (AUPRC) all exceeding 0.8 for both MRE detection and miRNA–target prediction (MRE prediction, Accuracy: 0.859, F1 score: 0.86, AUPRC: 0.927; miRNA–target prediction, Accuracy: 0.836, F1 score: 0.839, AUPRC: 0.902).

microT-CNN model architecture was further evaluated through ablation experiments on the same validation set. Distinct models were trained using each of the five “expert” branches incorporated into the microT-CNN core model individually to assess their specific performance. The highest performance was observed in the miRNA-MRE chimeric branch (AUC: 0.88), followed by the conservation branch (AUC: 0.729) and the miRNA-MRE duplex structure hybrid branch (AUC: 0.696) (Supplementary Fig. 2a). To assess the robustness of the microT-CNN architecture, we evaluated distinct models by combining the top-performing feature branches (miRNA-MRE chimeric + conservation, miRNA-MRE chimeric + duplex structure) or by removing the lowest-performing branch (RNA folding). Although the models still achieved high performance (AUC range: 0.898–0.918), microT-CNN outperformed all the others in accurately identifying genuine MREs (AUC: 0.922) (Supplementary Fig. 2b).

### microT-CNN identifies functional canonical and non-canonical interactions in 3′ UTR and coding regions

**Figure 3:**
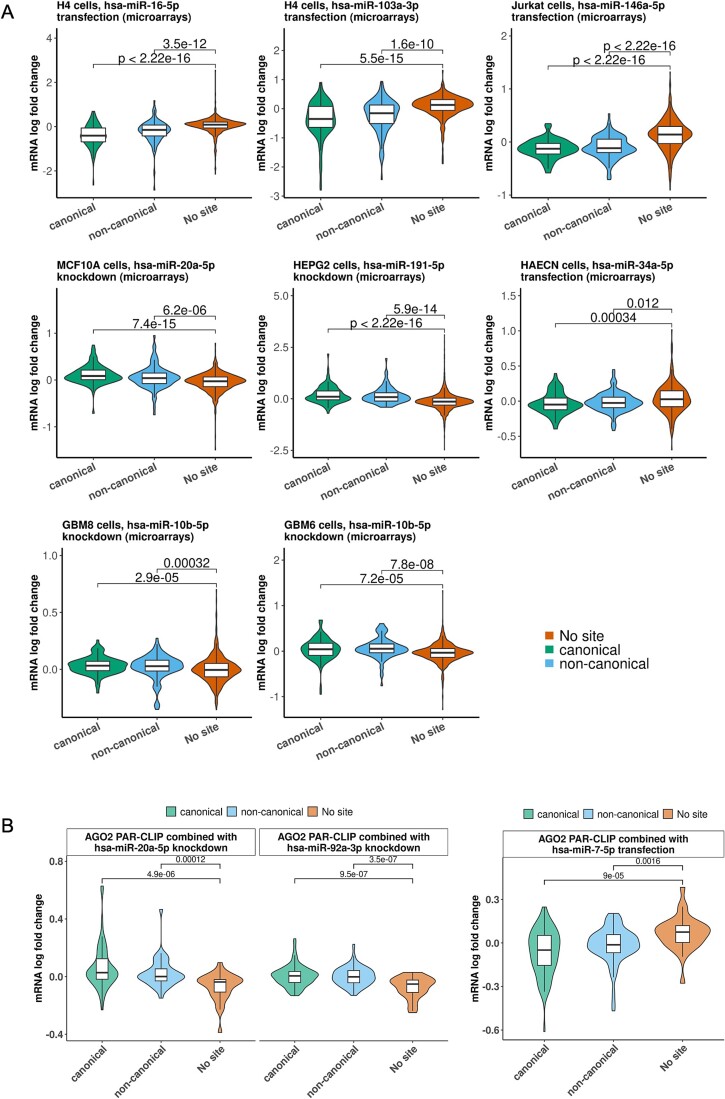
Functional efficacy of microT-CNN-detected (non-)canonical miRNA targets. (a) The functional efficacy of the predicted targets was examined in eight public gene expression profiling datasets following miRNA transfection or knockdown, corresponding to seven cell types. Response of the top 100 microT-CNN-scored targeted mRNAs to miRNA perturbation experiments was evaluated independently per tested cell type, experimental technique, and condition. Violin plots of mRNA fold changes in a log_2_ scale for miRNA targets comprising at least one predicted canonical MRE or supported only by non-canonical miRNA binding sites were compared to those that lack any site of the considered miRNAs. *P*-values for the differences in expression changes of the identified (non-)canonical miRNA targets compared to transcripts lacking any predicted miRNA binding site are portrayed (two-tailed Wilcoxon rank-sum test). (b) The functional efficacy of the predicted targets was examined in two AGO-PAR-CLIP HEK293 libraries combined with three gene expression profiling datasets following miRNA (left) knockdown or (right) transfection. The response of the microT-CNN-detected targeted mRNAs to the combined experiments was evaluated independently per miRNA. Boxplots of mRNA fold changes in a log_2_ scale for targets comprising at least one predicted canonical MRE or supported by only non-canonical miRNA binding sites were compared to those lacking any site of the considered miRNAs. *P*-values for the differences in expression changes of the identified (non-)canonical miRNA targets compared to transcripts lacking any predicted miRNA binding site are portrayed (two-tailed Wilcoxon rank-sum test).

To investigate the functional importance of the microT-CNN-derived canonical and non-canonical miRNA binding sites in 3′ UTR and CDS regions, we utilized eight public high-throughput gene expression profiling datasets following transfection or knockdown of specific miRNAs corresponding to six distinct cell types (GEO accessions: GSE42823, GSE21132, GSE33538, GSE22790, GSE22143, GSE68424, Supplementary Table 6). In the conducted comparisons, we measured miRNA–target fold changes in three distinct groups: (i) canonical miRNA targets with at least one binding site with perfect complementarity, (ii) miRNA targets participating in interactions resolved only by non-canonical binding sites, and (iii) transcripts lacking sites for the examined miRNAs (Fig. 3a, Supplementary Data Table). In all miRNA perturbation experiments, we observed that the top-scored microT-CNN detected miRNA targets (*n* = 100) were significantly downregulated or upregulated upon transfection or knockdown of different miRNAs compared to transcripts having no miRNA binding site (range of *P*-values canonical miRNA targets: 2.22 × 10^−16^–34 × 10^−5^, *P*-values non-canonical miRNA targets: 5.9 × 10^−14^–12 × 10^−3^, two-tailed Wilcoxon rank-sum test, 267 < *n*_no-site_ < 2889).

Due to the inherent limitations in identifying direct miRNA targets through functional experiments, we subsequently adopted an approach to assess the functional efficacy of direct microT-CNN-derived (non-)canonical miRNA targets. We combined the AGO-enriched regions of two PAR-CLIP HEK293 libraries (GEO accessions: GSE21918, Supplementary Table 8) with three gene expression profiling datasets following miRNA knockdown (GEO accessions: GSE21577, GSE46039) or transfection (GEO accessions: GSE14537) (Supplementary Table 6). Fold changes of (non-)canonical miRNA targets overlapping with AGO-enriched regions were evaluated against transcripts lacking sites for the examined miRNAs (Fig. 3b, Supplementary Data Table). In all comparisons, we observed that direct microT-CNN-detected targets were significantly downregulated or upregulated upon transfection or knockdown of different miRNAs compared to transcripts having no miRNA binding site (range of *P*-values canonical miRNA targets: 9.5 × 10^−7^–9 × 10^−5^, *P*-values non-canonical miRNA targets: 16 × 10^−4^–3.5 × 10^−7^, two-tailed Wilcoxon rank-sum test, 29 < *n*_no-site_ < 39).

While addressing the efficacy of the canonical and non-canonical miRNA binding sites is essential, an equally crucial aspect lies in deciphering the functional efficacy of miRNA–gene interactions retrieved from CDS regions. To this end, we utilized the aforementioned eight miRNA perturbation high-throughput gene expression profiling datasets (GEO accessions: GSE42823, GSE21132, GSE33538, GSE22790, GSE22143, GSE68424, Supplementary Table 6, Supplementary Data Table) to investigate the functional importance of the microT-CNN-derived miRNA targets from 3′ UTR and CDS regions. We measured target fold changes in miRNA targets with at least one binding site in the 3′ UTR region and miRNA targets resolved only by the CDS region (Supplementary Fig. 3). In the majority of the experiments, we observed that the top-scored microT-CNN-detected targets (*n* = 100) were significantly downregulated or upregulated upon transfection or knockdown of different miRNAs compared to transcripts having no miRNA binding site (range of *P*-values 3′ UTR miRNA targets: 3.6 × 10^−10^ − 0.2, *P*-values CDS miRNA targets: 0.0048–0.66, two-tailed Wilcoxon rank-sum test, 267 < *n*_no-site_ < 2889).

Across the various comparisons, regardless of the perturbation type and experiment, canonical and 3′ UTR miRNA targets showed a stronger association with more responsive genes. Still, a significant number of functional non-canonical and CDS miRNA targets corresponding to diverse cell types were detected in most of the experiments.

### microT-CNN accurately identifies experimentally supported virus-encoded microRNA interactions

Virus-encoded miRNA interactions are core elements by which viruses manipulate host gene expression to facilitate their replication, evade host immune responses, and promote viral pathogenesis [2, 3]. We investigated the performance of microT-CNN to identify not only host miRNA interactions but also virus-encoded miRNA–host target interactions and their respective miRNA binding sites. We utilized for the first time a *bona fide* set of 2789 EBV- and KSHV-derived (q) CLASH-supported miRNA binding sites (Materials and Methods, Supplementary Data Table), corresponding to 2243 virus-encoded miRNA–gene interactions derived from 23 viral miRNAs.

**Figure 4:**
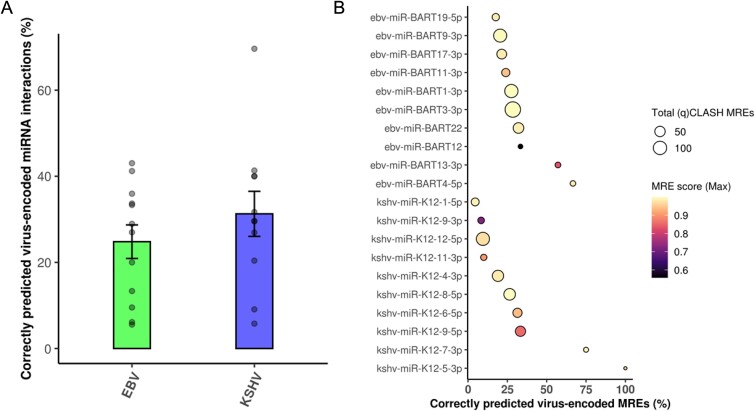
Evaluation of virus-encoded microT-CNN-detected miRNA targets with (q)CLASH experimental data. The utilized validation set is composed of 2966 unique EBV and KSHV-encoded miRNA binding sites, corresponding to 2331 virus-encoded miRNA–target interactions derived from 23 EBV and KSHV miRNAs. (a) The number of correctly predicted interactions (microT-CNN score > 0.7) versus the total (q)CLASH retrieved target pairs is portrayed for the distinct EBV- and KSHV-encoded miRNA interactions. (b) Dotplot representing the number of correctly predicted virally encoded MREs (vMREs) versus the total (q)CLASH MREs. The size of the dot portrays the total (q)CLASH vMREs per miRNA. The top MRE score is displayed per distinct viral miRNA in a black-to-yellow color gradient.

microT-CNN accurately predicts ~50% EBV-/KSHV-derived genuine miRNA–host interactions (microT-CNN score > 0.5), 1145 in total, and 30% highly scored (microT-CNN score > 0.7), 700 in total, across all the 23 virus-encoded miRNAs, capturing up to 67% in specific cases (Fig. 4a**)**. The model also performed well in investigating the correct miRNA binding sites. Specifically, microT-CNN accurately identifies 204 out of the 846 provided viral miRNA–host MREs, detecting at least one per virus-encoded miRNA. In miRNA cases with few provided genuine MREs (<5), the model captures >70% miRNA binding sites (Fig. 4b).

### Evaluation of microT-CNN against other *de novo* models

**Figure 5:**
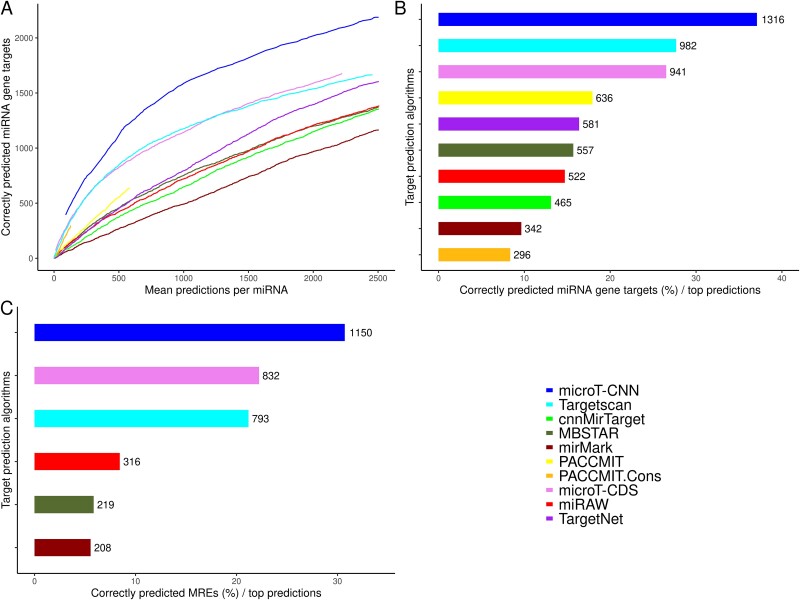
Evaluation of microT-CNN performance against state-of-the-art implementations. The utilized validation set comprises 3743 unique MREs, corresponding to 3548 miRNA–target interactions derived from 153 miRNAs supported by chimeric miRNA–gene fragments. (a) The number of correctly predicted miRNA targets for each implementation is plotted versus the mean predictions per miRNA at different cutoff thresholds for the different models. (b) The percentage of the correctly predicted miRNA–gene interactions versus the 100 000 top-scored predictions per model is displayed. The actual number of correctly predicted miRNA–gene interactions is displayed per model. (c) The percentage of the correctly predicted MREs at the 100 000 top-scored miRNA–gene predictions per model is portrayed. The actual number of correctly predicted MREs is displayed per model.

To assess microT-CNN accuracy and to estimate the information gained with the incorporation of non-canonical and CDS miRNA binding sites, we compared our model against state-of-the-art *de novo* miRNA target models, including microT-CDS [10], Targetscan v7 [9], MBSTAR [13], mirMark [14], PACCMIT-CDS [12] (with and without conservation feature), and recent developed deep learning applications, miRAW [20], cnnMirTarget [21], and TargetNet [19]. A significant aspect of the *de novo* miRNA target models is their efficiency in correctly determining *bona fide* miRNA targets at a low number of total predictions per miRNA. Therefore, we evaluated the distinct models against a validation set of experimentally verified, direct miRNA targets derived from chimeric miRNA–target fragments. The validation set comprised 3743 unique miRNA binding sites corresponding to 3548 miRNA–target interactions derived from 153 miRNAs (Supplementary Table 2, Supplementary Data Table). The number of correctly predicted MREs per tested *in silico* method is compared against the total predictions per miRNA for different score thresholds, accompanied by the amount of correctly predicted targets for the top-scored predicted miRNA–target pairs per model (*n* = 100 000, Fig. 5a and b). Different cutoff thresholds were also applied for the top-scored predicted miRNA–target pairs (i.e. 5000, 10 000, and 50 000) to assess the robustness of our analysis (Supplementary Fig. 4). A separate comparison capturing algorithms’ efficiency to predict correct miRNA binding sites in the top-scored miRNA targets was also conducted (Fig. 5c, Supplementary Fig. 4). Only the models providing this level of information are included in this analysis. microT-CNN exhibits a greater ability to discriminate miRNA interactions at equivalent numbers of total predictions, providing 1.2–1.4-fold more miRNA–gene interactions and 1.07–1.4 more MREs, corresponding to a significantly higher sensitivity both at the gene and binding site level (Fig. 5b and c, Supplementary Fig. 4).

A similar analysis was also performed in a separate validation set derived from the analysis of two AGO-CLIP-seq datasets (GEO/SRA accession: GSE59944, SRR359787, Supplementary Data Table) from two distinct cell types (C8166, hESC). The validation set comprised 13 357 miRNA binding events derived from 110 miRNAs, corresponding to 12 343 unique miRNA–target pairs. microT-CNN, Targetscan, MBSTAR, and microT-CDS performed similarly at the miRNA–gene level. Still, microT-CNN presented a significantly higher performance at the miRNA binding level, providing 10% more correctly predicted miRNA binding events at the top-scored targets (*n* = 100 000, Supplementary Fig. 5).

**Figure 6:**
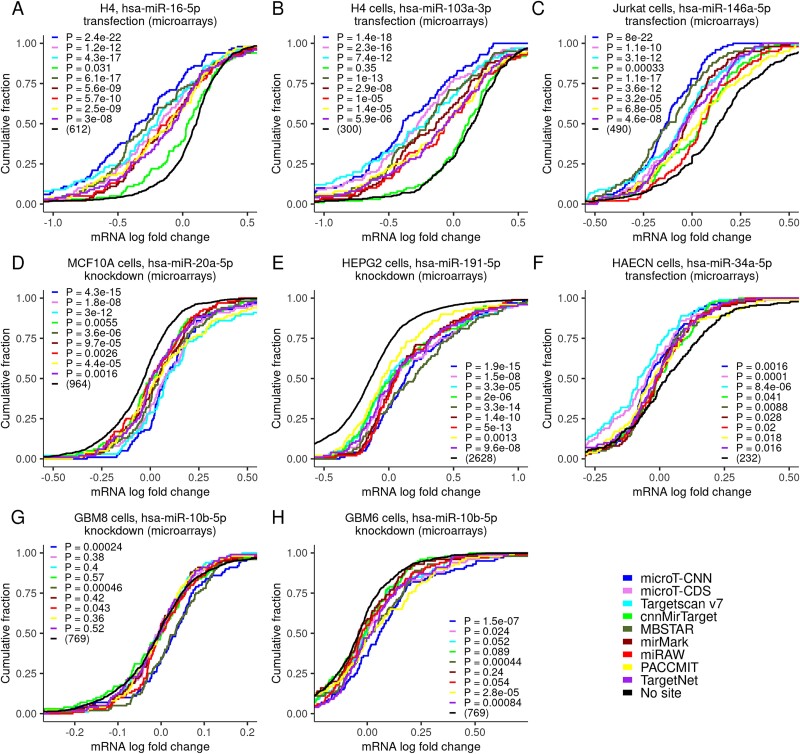
Evaluation of the functional efficacy of the microT-CNN-detected miRNA targets against state-of-the-art implementations. The response of targeted mRNAs to miRNA perturbation experiments was evaluated independently per tested cell type, experimental technique, and condition for the different models (a–h). Cumulative distributions of mRNA fold changes in a log_2_ scale for targets comprising at least one predicted MRE in the CDS or 3′ UTR regions were compared to those that lacked any site of the considered miRNAs (one-sided Kolmogorov–Smirnov test). Functional efficacy was assessed for the top 100 predictions per model. Implementations that lack any target for the examined miRNAs were excluded from each comparison.

A significant aspect of the *de novo* miRNA–target implementations, aside from their ability to accurately detect genuine miRNA–binding events with low false positive rates, is to provide functionally relevant miRNA interactions. To this end, an extra evaluation was implemented using eight public high-throughput gene expression profiling datasets following the transfection or knockdown of specific miRNAs corresponding to seven distinct cell types (Fig. 6, Supplementary Table 6, Supplementary Data Table). To ascertain an impartial evaluation, cumulative distributions of fold changes were compared for equivalent sets of top 100 predicted targets, i.e. genes with one or more predicted MRE, against genes lacking any site(s) for the considered miRNAs. microT-CNN exerted significant differences in expression changes compared to transcripts lacking any predicted binding site (range of *P*-values (a–h): 8 × 10^−22^–16 × 10^−4^, one-sided Kolmogorov–Smirnov test, 232 < *n*_no-site_ < 2628). Compared to the other *de novo* implementations, microT-CNN displayed the greatest miRNA–target effectiveness in most cases (range of *P*-values (a–e, h): 1.5 × 10^−7^–2.4 × 10^−22^, one-sided Kolmogorov–Smirnov test; Fig. 6a–e, h). In Fig. 6f and g, it performed similarly to, or less favorably than, Targetscan and MBSTAR, respectively, and better than the rest of the implementations (range of *P*-values (f): 0.0001; 0.0016, range of *P*-values (g): 0.00024; 0.00046, one-sided Kolmogorov–Smirnov test).

## Discussion and Conclusion

miRNAs are crucial post-transcriptional gene expression regulators controlling various cellular processes in healthy and/or diseased states [1]. In addition to host miRNAs, efforts have been made to understand virally encoded miRNAs and their interactions with host targets to elucidate their role in viral pathogenesis and replication [2, 3]. Deciphering miRNA targets is paramount to understanding their function, which now counts more than two decades of effort [6]. Several experimental techniques and computational tools have been developed in this endeavor; however, the complexity of the existing direct high-throughput experimental techniques and the low performance of most existing models leaves the miRNA interactome incomplete and miRNA functions partially elucidated [3]. By leveraging large-scale experimental data and recent advancements in machine learning, alongside computational docking for compatibility and stability, we can significantly improve the accuracy of miRNA–target interaction identification [41].

Here, we introduce microT-CNN, an avant-garde next-generation DCNN framework designed to address the complexity of miRNA targeting and provide the whole miRNA interactome spectrum. By leveraging a multilayered CNN design and incorporating data from over 60 000 miRNA binding sites from direct AGO-CLIP-seq experiments, chimeric miRNA–target fragments, and >70 tissue-matched miRNA perturbation experiments, microT-CNN accurately identifies (non-)canonical miRNA binding sites, within the 3′ UTR and CDS regions. The multi-agent CNN framework learns hidden miRNA binding patterns within the MRE regions, the miRNA-binding structure, the RNA accessibility, and the conservation of the miRNA-targeted region. This approach facilitates the extraction of informative, hidden patterns separately in 3′ UTR and CDS regions and detects both canonical and non-canonical miRNA binding sites, including previously neglected 3′ compensatory miRNA pairings, capturing the whole spectrum of interactions. miRNA binding sites from 3′ UTR and CDS regions are aggregated in a GBM meta-learner, trained on miRNA targets derived from miRNA transfection experiments to predict functional targets.

microT-CNN provides >70% direct miRNA targets on a *bona fide* test set of ~3500 miRNA–target interactions, corresponding to up to 1.4-fold more validated miRNA targets and miRNA binding sites than leading implementations. The model’s ensemble multilayer design provides a robust and precise tool for predicting host and virus-encoded miRNA targets. microT-CNN accurately detects up to 67% of genuine EBV- and KSHV-derived miRNA–gene interactions and one out of four MREs of viral miRNAs when compared for the first time on ~3000 (q)CLASH-derived genuine viral-encoded miRNA interactions, holding promise for elucidating the role of viral-encoded miRNAs in modulating host gene expression. These represent significant breakthroughs in miRNA–target prediction, shedding light on previously unexplored facets of the miRNA interactome. In future directions, continuous advancements in high-quality, direct, high-throughput experiments can be leveraged to retrain miRNA–target detection models, improving their accuracy in identifying functional miRNA targets beyond current implementations. Moreover, future model upgrades could integrate experiment-specific expression data, allowing the framework to capture context-dependent miRNA activity and diverse regulatory networks across disease-, tissue-, and patient-specific contexts.

**Key Points**

- microT-CNN is a multilayer Deep Convolutional Neural Network framework that predicts functional (non-)canonical seed-based and 3′ compensatory miRNA binding events on 3′UTR and coding regions.
- microT-CNN was deployed by integrating an extensive set of >60 000 miRNA binding events and ~30 000 tissue-matched functional miRNA–gene target interactions across 26 different cell types from ~130 AGO-CLIP-seq experiments and ~70 miRNA perturbation experiments.
- microT-CNN is the first model to be evaluated on ~3000 genuine virus-encoded miRNA binding sites, and it predicts up to 67% of miRNA binding events, corresponding to one out of four virus-encoded miRNA target sites.
- microT-CNN provides >70% direct miRNA targets on a comprehensive test set of genuine miRNA binding events and detects 1.4-fold more validated miRNA binding sites than leading implementations.

## Supplementary Material

Supplement_bbae678

Supplementary_Data_Table_bbae678

## Data Availability

All data used in the study are available in supplementary materials. The microT-CNN is available on GitHub [https://github.com/dianalabgr/microT-CNN.git].
