# Supplementary material for: microT-CNN: an avant-garde deep convolutional neural network unravels functional miRNA targets beyond canonical sites
Source: Brief Bioinform. 2024 Dec 31;26(1):bbae678. doi: 10.1093/bib/bbae678 (PMC11685103; doi:10.1093/bib/bbae678)
Supplement: Supplement_bbae678 [file supplement_bbae678.docx]

**Supplementary Information:**

**microT-CNN: An avant-garde Deep Convolutional Neural Network unravels functional miRΝΑ targets beyond canonical sites**

**Supplementary Table 1: Summary of the cell type/tissue associations for the extraction of miRNA-targeted regions incorporated in training/test sets.**

| **Tissue** | **Cell Type** | **AGO-CLIP-Seq libraries** | **miRNA perturbation experiments** | |
| --- | --- | --- | --- | --- |
| **Kidney** | HEK293, HEK293T, 293S | 22 | 5 | |
| **B lymphocyte** | CRL1432, CRL1596, H929, P3HR1, DG75, ST486, BC1, BC3 | 7 | 5 |  |
| **Bone Marrow** | HMSC, BCBL1, HS27A, HS5, TRHBMEC | 18 | 1 | |
| **Pancreas** | PANC1, BETA | 1 | 2 | |
| **Brain** | H4, SW1783, U87, U872M1 | 11 | 7 | |
| **Mammary Gland** | MCF7, MCF7FR, MDAMB231, SUM159, BT474 | 13 | 7 | |
| **Cervix** | HELA, TZMBL | 3 | 30 | |
| **Ovary** | HEY, A2780 | 4 | 3 | |
| **Prostate** | LNCAP, PC3, DU145, 22RV1, LAPC4 | 5 | 5 | |
| **Intestine** | HCT116 | 36 | 2 | |

**Supplementary Table 2: Summary of training/test set utilized for the deployment of the microT-CNN-MRE detection model.**

|  | **miRNAs in interactions** | | | **Genes in interactions** | | **miRNA-target binding events** | |
| --- | --- | --- | --- | --- | --- | --- | --- |
|  | **Training** | | **Test** | **Training** | **Test** | **Training** | **Test** |
| **Positive Instances** | | | | | | | |
|  | | | | | | | |
| ***miRNA-mRNA chimeric fragments*** | 347 | 222 | | 4,249 | 1,933 | 10,518 | 3,848 |
| ***AGO-CLIP/miRNA perturbation experiments*** | 54 | 128 | | 1,422 | 190 | 8,210 | 287 |
| **Negative Instances** | | | | | | | |
| ***Background CLIP-Seq*** | 392 | 222 | | 1,296 | 877 | 7,542 | 1,230 |
| ***AGO-CLIP/miRNA perturbation experiments*** | 55 | 40 | | 1,034 | 408 | 1,700 | 605 |
| ***miRNA perturbation experiments*** | 56 | 37 | | 1,295 | 521 | 1,938 | 674 |

**Supplementary Table 3: Summary of miRNA-target binding sites on 3’ UTR and CDS regions utilized in the microT-CNN-MRE detection model.**

| **Biotype** | **Positive set** | | **Negative set** | |
| --- | --- | --- | --- | --- |
|  | **Training** | **Test** | **Training** | **Test** |
| **UTR3** | 10,197 | 2,323 | 21,485 | 2,400 |
| **CDS** | 8,531 | 2,014 | 18,051 | 2,378 |

**Supplementary Table 4: Summary of miRNA-target interactions on 3’ UTR and CDS regions utilized in the microT-CNN-target GBM detection model.**

| **Biotype** | **Positive set** | **Negative set** |
| --- | --- | --- |
| **UTR3** | 1,072 | 950 |
| **CDS** | 1,041 | 1,059 |

**Supplementary Table 5: Description of the binding types supported by microT-CNN.**

| **Binding Type** | **Description** |
| --- | --- |
| **9mer 3prime** | 9mer canonical with 3′ supplementary binding |
| **9mer** | 9mer canonical |
| **9mer GU** | base pairing in 1-9 positions with a GU wobble pair |
| **9mer nonCanonical** | base pairing in 1-9 positions with a target bulge and/or a GU wobble pair |
| **8mer 3prime** | 8mer/8mer1A canonical with 3′ supplementary binding |
| **8mer** | 8mer canonical |
| **8mer1A** | 7mer canonical with additional A in position 1 |
| **8mer GU** | base pairing in 1-8 or 2-9 positions with a GU wobble pair |
| **8mer nonCanonical** | base pairing in 1-9 positions with mismatch or miRNA bulge and/or a target bulge and/or a GU wobble pair |
| **7mer 3prime** | 7mer/7mer1A canonical with 3′ supplementary binding |
| **7mer** | 7mer canonical |
| **7mer1A** | 6mer canonical with an additional A in position 1 |
| **7mer GU** | base pairing in 2-8 positions with a GU wobble pair |
| **7mer nonCanonical** | base pairing in 1-8 positions with a mismatch or miRNA bulge and/or a target bulge |
| **6mer 3prime** | 6mer canonical with 3′ supplementary binding |
| **6mer** | 6mer canonical |
| **offset6mer** | 6mer base pairing in 3-8 positions |
| **6mer nonCanonical 3prime** | base pairing in 2-8 positions with a mismatch or miRNA bulge and/or a target bulge, with 3′ supplementary binding |
| **centered** | base pairing in 4-15 positions with at least 8 consecutive matches |
| **imperfect centered** | base pairing in 4-15 positions with at least 8 matches and/or less than 2 GU wobble pairs |

**Supplementary Table 6: Summary of the collected microarray experiments in human species upon specific miRNA deregulation. The datasets were utilized to extract independent training and test sets of positive and negative miRNA binding sites for microT-CNN deployment.**

| **Accession** | **Repository** | **Authors** | **Cell line** | **microRNA** | **microRNA treatment** | **Post-Transfection Cell Harvest Time/Experimental Condition** |
| --- | --- | --- | --- | --- | --- | --- |
| GSE12278 | ncbi.nlm.nih.gov/geo | Sander et al. | CRL1432 | hsa-miR-26a-5p | Overexpression | 72h |
| GSE12278 | ncbi.nlm.nih.gov/geo | Sander et al. | CRL1596 | hsa-miR-26a-5p | Overexpression | 72h |
| GSE35948 | ncbi.nlm.nih.gov/geo | Misiewicz-Krzeminska et al. | H929 | hsa-miR-214-3p | Overexpression | - |
| GSE56268 | ncbi.nlm.nih.gov/geo | Schneider et al. | P3HR1 | hsa-miR-28-5p | Overexpression | 12h |
| GSE56268 | ncbi.nlm.nih.gov/geo | Schneider et al. | P3HR1 | hsa-miR-28-5p | Overexpression | 24h |
| GSE42823 | ncbi.nlm.nih.gov/geo | Nelson et al. | H4 | hsa-miR-15b-3p | Overexpression | 48h |
| GSE42823 | ncbi.nlm.nih.gov/geo | Nelson et al. | H4 | hsa-miR-195-5p | Overexpression | 48h |
| GSE42823 | ncbi.nlm.nih.gov/geo | Nelson et al. | H4 | hsa-miR-320b | Overexpression | 48h |
| GSE42823 | ncbi.nlm.nih.gov/geo | Nelson et al. | H4 | hsa-miR-16-5p | Overexpression | 48h |
| GSE42823 | ncbi.nlm.nih.gov/geo | Nelson et al. | H4 | hsa-miR-103a-3p | Overexpression | 48h |
| GSE21132 | ncbi.nlm.nih.gov/geo | Li et al. | Jurkat | hsa-miR-146a-5p | Overexpression | 48h |
| GSE33538 | ncbi.nlm.nih.gov/geo | Bossel Ben-Moshe et al. | MCF10A | hsa-miR-20a-5p | Silencing | 2h post-EGF stimulation |
| GSE22790 | ncbi.nlm.nih.gov/geo | Elyakim et al. | HEPG2 | hsa-miR-191-5p | Anti-miR | - |
| GSE22143 | ncbi.nlm.nih.gov/geo | Marcet et al. | HAEC | hsa-miR-34a-5p | Overexpression | 48h |
| GSE68424 | ncbi.nlm.nih.gov/geo | Teplyuk et al. | GBM8 | hsa-miR-10b-5p | Inhibition | 24h |
| GSE34482 | ncbi.nlm.nih.gov/geo | Choudhury et al. | SW1783 | hsa-miR-376a-5p | Overexpression | 24h |
| GSE34482 | ncbi.nlm.nih.gov/geo | Choudhury et al. | U87 | hsa-miR-376a-5p | Overexpression | 24h |
| GSE19693 | ncbi.nlm.nih.gov/geo | Chen et al. | U87 | hsa-miR-20a-5p | Overexpression | - |
| GSE35170 | ncbi.nlm.nih.gov/geo | Lin et al. | U87-2M1 | hsa-miR-10b-5p | Inhibition | - |
| - | psilac.mdc-berlin.de | Selbach et al. | HELA | hsa-miR-155-5p | Overexpression | 8h |
| - | psilac.mdc-berlin.de | Selbach et al. | HELA | hsa-miR-155-5p | Overexpression | 32h |
| - | psilac.mdc-berlin.de | Selbach et al. | HELA | hsa-miR-16-5p | Overexpression | 8h |
| - | psilac.mdc-berlin.de | Selbach et al. | HELA | hsa-miR-16-5p | Overexpression | 32h |
| GSE18625 | ncbi.nlm.nih.gov/geo | Gregersen et al. | DLD1 | hsa-miR-145-5p | Overexpression | 24h |
| GSE7754 | ncbi.nlm.nih.gov/geo | Chang et al. | HCT116 | hsa-miR-34a-5p | Overexpression | 2w after retroviral infection |
| GSE51875 | ncbi.nlm.nih.gov/geo | Lee et al. | HCT116 | hsa-miR-147a | Overexpression | 3d |
| GSE37596 | ncbi.nlm.nih.gov/geo | Hwang et al. | HT29 | hsa-miR-146a-5p | Overexpression | 2w after lentiviral infection |
| GSE21901 | ncbi.nlm.nih.gov/geo | Hollander et al. | HEK293 | hsa-miR-212-3p | Overexpression | - |
| GSE35620 | ncbi.nlm.nih.gov/geo | Hu et al. | HEK293 | hsa-miR-941 | Overexpression | 24h |
| GSE35620 | ncbi.nlm.nih.gov/geo | Hu et al. | HEK293T | hsa-miR-941 | Overexpression | 24h |
| GSE58142 | ncbi.nlm.nih.gov/geo | Frankel et al. | MCF7 | hsa-miR-95a-3p | Overexpression | 24h |
| GSE31397 | ncbi.nlm.nih.gov/geo | Frankel et al. | MCF7 | hsa-miR-101-3p | Overexpression | 24h |
| GSE19777 | ncbi.nlm.nih.gov/geo | Rao et al. | MCF7FR | hsa-miR-221-3p | Silencing | 72h |
| GSE40058 | ncbi.nlm.nih.gov/geo | Luo et al. | MDAMB231 | hsa-miR-200c-3p | Overexpression | - |
| GSE40058 | ncbi.nlm.nih.gov/geo | Luo et al. | MDAMB231 | hsa-miR-205-5p | Overexpression | - |
| GSE40058 | ncbi.nlm.nih.gov/geo | Luo et al. | MDAMB231 | hsa-mir-375 | Overexpression | - |
| GSE50697 | ncbi.nlm.nih.gov/geo | Taube et al. | SUM159 | hsa-miR-203a-3p | Overexpression | - |
| GSE56967 | ncbi.nlm.nih.gov/geo | Hill et al. | HEY | hsa-miR-429 | Overexpression | 48h |
| GSE27431 | ncbi.nlm.nih.gov/geo | Shahab et al. | HEY | hsa-miR-128-3p | Overexpression | 48h |
| GSE27431 | ncbi.nlm.nih.gov/geo | Shahab et al. | HEY | hsa-miR-7-5p | Overexpression | 48h |
| GSE51053 | ncbi.nlm.nih.gov/geo | Kristensen et al. | DU145 | hsa-miR-224-5p | Overexpression | 48h |
| GSE31620 | ncbi.nlm.nih.gov/geo | Hudson et al. | LNCAP | hsa-miR-206 | Overexpression | 24h |
| GSE31620 | ncbi.nlm.nih.gov/geo | Hudson et al. | LNCAP | hsa-miR-27b-3p | Overexpression | 24h |
| GSE51053 | ncbi.nlm.nih.gov/geo | Kristensen et al. | PC3 | hsa-miR-224-5p | Overexpression | 48h |
| GSE51053 | ncbi.nlm.nih.gov/geo | Kristensen et al. | PC3 | hsa-miR-452-5p | Overexpression | 48h |
| GSE25215 | ncbi.nlm.nih.gov/geo | Ikeda et al. | MIA PaCa-2 | hsa-miR-193b-3p | Overexpression | Microarrays |
| GSE40189 | ncbi.nlm.nih.gov/geo | Ouyang et al. | PANC1 | hsa-miR-10b-5p | Overexpression | 20h |
| GSE86432 | ncbi.nlm.nih.gov/geo | Dzikiewicz-Krawczyk et al. | ST486 | hsa-miR-150-5p | Overexpression | Microarrays |
| GSE19232 | ncbi.nlm.nih.gov/geo | Tome et al. | HMSC | hsa-miR-335-5p | Overexpression | 9d |
| GSE21577 | ncbi.nlm.nih.gov/geo | Hafner et al | HEK293 | hsa-miR-20a-5p | Inhibition | 20h |
| GSE46039 | ncbi.nlm.nih.gov/geo | Helwak et al | HEK293 | hsa-miR-92a-3p | Inhibition | 48h |
| GSE14537 | ncbi.nlm.nih.gov/geo | Hafner et al | HEK293 | hsa-miR-7-5p | Overexpression | 20h |

**Supplementary Table 7: Summary of the collected RNA Sequencing experiments in human species upon miRNA overexpression. The datasets were utilized to extract independent training and test sets of positive and negative miRNA binding sites for microT-CNN deployment.**

| **Accession** | **Repository** | **Authors** | **Cell line** | **microRNA** | **microRNA treatment** | **Post-Transfection Cell Harvest Time/Experimental Condition** |
| --- | --- | --- | --- | --- | --- | --- |
| GSE52531 | ncbi.nlm.nih.gov/geo | Nam et al.. | HEK293 | hsa-miR-124-3p | Overexpression | 24h |
| GSE52531 | ncbi.nlm.nih.gov/geo | Nam et al.. | HEK293 | hsa-miR-155-5p | Overexpression | 24h |
| GSE52531 | ncbi.nlm.nih.gov/geo | Nam et al.. | HELA | hsa-miR-155-5p | Overexpression | 24h |
| GSE52531 | ncbi.nlm.nih.gov/geo | Nam et al.. | HELA | hsa-miR-124-3p | Overexpression | 24h |
| GSE64615 | ncbi.nlm.nih.gov/geo | Polioudakis et al.. | HELA | hsa-miR-494-5p | Overexpression | - |
| GSE124530 | ncbi.nlm.nih.gov/geo | Liu W et al.. | HELA | hsa-let-7c-5p | Overexpression | 24h |
| GSE124530 | ncbi.nlm.nih.gov/geo | Liu W et al.. | HELA | hsa-miR-107 | Overexpression | 24h |
| GSE124530 | ncbi.nlm.nih.gov/geo | Liu W et al.. | HELA | hsa-miR-10a-5p | Overexpression | 24h |
| GSE124530 | ncbi.nlm.nih.gov/geo | Liu W et al.. | HELA | hsa-miR-124-3p | Overexpression | 24h |
| GSE124530 | ncbi.nlm.nih.gov/geo | Liu W et al.. | HELA | hsa-miR-126-3p | Overexpression | 24h |
| GSE124530 | ncbi.nlm.nih.gov/geo | Liu W et al.. | HELA | hsa-miR-126-5p | Overexpression | 24h |
| GSE124530 | ncbi.nlm.nih.gov/geo | Liu W et al.. | HELA | hsa-miR-133b | Overexpression | 24h |
| GSE124530 | ncbi.nlm.nih.gov/geo | Liu W et al.. | HELA | hsa-miR-142-3p | Overexpression | 24h |
| GSE124530 | ncbi.nlm.nih.gov/geo | Liu W et al.. | HELA | hsa-miR-145-5p | Overexpression | 24h |
| GSE124530 | ncbi.nlm.nih.gov/geo | Liu W et al.. | HELA | hsa-miR-146a-5p | Overexpression | 24h |
| GSE124530 | ncbi.nlm.nih.gov/geo | Liu W et al.. | HELA | hsa-miR-155-5p | Overexpression | 24h |
| GSE124530 | ncbi.nlm.nih.gov/geo | Liu W et al.. | HELA | hsa-miR-15a-5p | Overexpression | 24h |
| GSE124530 | ncbi.nlm.nih.gov/geo | Liu W et al.. | HELA | hsa-miR-16-5p | Overexpression | 24h |
| GSE124530 | ncbi.nlm.nih.gov/geo | Liu W et al.. | HELA | hsa-miR-17-5p | Overexpression | 24h |
| GSE124530 | ncbi.nlm.nih.gov/geo | Liu W et al.. | HELA | hsa-miR-193b-3p | Overexpression | 24h |
| GSE124530 | ncbi.nlm.nih.gov/geo | Liu W et al.. | HELA | hsa-miR-200a-3p | Overexpression | 24h |
| GSE124530 | ncbi.nlm.nih.gov/geo | Liu W et al.. | HELA | hsa-miR-200b-3p | Overexpression | 24h |
| GSE124530 | ncbi.nlm.nih.gov/geo | Liu W et al.. | HELA | hsa-miR-200c-3p | Overexpression | 24h |
| GSE124530 | ncbi.nlm.nih.gov/geo | Liu W et al.. | HELA | hsa-miR-206 | Overexpression | 24h |
| GSE124530 | ncbi.nlm.nih.gov/geo | Liu W et al.. | HELA | hsa-miR-210-3p | Overexpression | 24h |
| GSE124530 | ncbi.nlm.nih.gov/geo | Liu W et al.. | HELA | hsa-miR-21-5p | Overexpression | 24h |
| GSE124530 | ncbi.nlm.nih.gov/geo | Liu W et al.. | HELA | hsa-miR-31-5p | Overexpression | 24h |
| GSE124530 | ncbi.nlm.nih.gov/geo | Liu W et al.. | HELA | hsa-miR-34a-5p | Overexpression | 24h |
| GSE124530 | ncbi.nlm.nih.gov/geo | Liu W et al.. | HELA | hsa-miR-9-3p | Overexpression | 24h |
| GSE124530 | ncbi.nlm.nih.gov/geo | Liu W et al.. | HELA | hsa-miR-9-5p | Overexpression | 24h |
| GSE88757 | ncbi.nlm.nih.gov/geo | Ottaviani S et al.. | PANC1 | hsa-miR-125b | Overexpression | 24h. |

**Supplementary Table 8: Summary of the collected AGO-PAR-CLIP and AGO-HITS-CLIP experiments in human species. The AGO signal from these datasets was utilized to extract independent training and test sets of positive and negative miRNA binding sites for microT-CNN deployment.**

| **Accession** | **Repository** | **Authors** | **Experiment** | **Species** | **Cell line** | **Samples** |
| --- | --- | --- | --- | --- | --- | --- |
| GSE44404 | ncbi.nlm.nih.gov/geo | Fedor V | HITS-CLIP | human | 293S | GSM1084041,  GSM1084043, GSM1084047,  GSM1084065, GSM1084079,  GSM1084040, GSM1084042,  GSM1084044,  GSM1084046, GSM1084064 |
| GSE32109 | ncbi.nlm.nih.gov/geo | Gottwein E | PAR-CLIP | human | BC1 | GSM796038 |
| GSE32109 | ncbi.nlm.nih.gov/geo | Gottwein E | PAR-CLIP | human | BC3 | GSM796040 |
| GSE41357 | ncbi.nlm.nih.gov/geo | Haecker I | HITS-CLIP | human | BC3 | GSM1015450, GSM1015451, GSM1015452 |
| GSE41357 | ncbi.nlm.nih.gov/geo | Haecker I | HITS-CLIP | human | BCBL1 | GSM1015453, GSM1015454 |
| GSE43909 | ncbi.nlm.nih.gov/geo | Erhard F | PAR-CLIP | human | BCBL1 | GSM1074233, GSM1074234 |
| GSE43909 | ncbi.nlm.nih.gov/geo | Erhard F | PAR-CLIP | human | DG75 | GSM1074231, GSM1074232 |
| GSE52314 | ncbi.nlm.nih.gov/geo | Kameswaran1 V | HITS-CLIP | human | BETA | GSM1262771 |
| GSE52084 | ncbi.nlm.nih.gov/geo | Boudreau RL | HITS-CLIP | human | brain | GSM1259105, GSM1259106, GSM1259107, GSM1259108, GSM1259109, GSM1259110, GSM1259111, GSM1259112, GSM1259113, GSM1259114,  GSM1259115 |
| GSE57855 | ncbi.nlm.nih.gov/geo | Pillai MM | HITS-CLIP | human | BT474 | GSM1395166, GSM1395167, GSM1395168 |
| GSE57855 | ncbi.nlm.nih.gov/geo | Pillai MM | HITS-CLIP | human | MCF7 | GSM1395163, GSM1395164, GSM1395165 |
| GSE57855 | ncbi.nlm.nih.gov/geo | Pillai MM | HITS-CLIP | human | MDAMB231 | GSM1395169, GSM1395170, GSM1395171 |
| GSE28865 | ncbi.nlm.nih.gov/geo | Kishore S | PAR-CLIP | human | HEK293 | GSM714644, GSM714645, GSM714646, GSM714647 |
| GSE21918 | ncbi.nlm.nih.gov/geo | Hafner M | PAR-CLIP | human | HEK293 | GSM545212, GSM545213, GSM545214, GSM545215 |
| GSE43574 | ncbi.nlm.nih.gov/geo | Memczak S | PAR-CLIP | human | HEK293 | GSM1065667, GSM1065668, GSM1065669, GSM1065670 |
| GSE42701 | ncbi.nlm.nih.gov/geo | Xue Y | HITS-CLIP | human | HELA | GSM1048187 |
| GSE41272 | ncbi.nlm.nih.gov/geo | Balakrishnan I | HITS-CLIP | human | HMSC | GSM1013110, GSM1013113, GSM1013114, GSM1013115, GSM1013116 |
| GSE41272 | ncbi.nlm.nih.gov/geo | Balakrishnan I | HITS-CLIP | human | HS27A | GSM1013103, GSM1013106, GSM1013109 |
| GSE41272 | ncbi.nlm.nih.gov/geo | Balakrishnan I | HITS-CLIP | human | HS5 | GSM1013104, GSM1013105, GSM1013108 |
| GSE41272 | ncbi.nlm.nih.gov/geo | Balakrishnan I | HITS-CLIP | human | TRHBMEC | GSM1013112, GSM1013117, GSM1013118 |
| GSE41437 | ncbi.nlm.nih.gov/geo | Skalsky RL | PAR-CLIP | human | EF3DAGO2 | GSM1020021 |
| GSE41437 | ncbi.nlm.nih.gov/geo | Skalsky RL | PAR-CLIP | human | LCL35 | GSM1020022 |
| GSE41437 | ncbi.nlm.nih.gov/geo | Skalsky RL | PAR-CLIP | human | LCLBAC | GSM1020023 |
| GSE41437 | ncbi.nlm.nih.gov/geo | Skalsky RL | PAR-CLIP | human | LCLBACD1 | GSM1020024 |
| GSE41437 | ncbi.nlm.nih.gov/geo | Skalsky RL | PAR-CLIP | human | LCLBACD3 | GSM1020025 |
| SRP075075 | ncbi.nlm.nih.gov/sra | Hamilton MP | PAR-CLIP | human | LNCAP | SRR3502923, SRR3502926, SRR3502931 |
| SRP075075 | ncbi.nlm.nih.gov/sra | Hamilton MP | PAR-CLIP | human | 22RV1 | SRR3502967, SRR3502969, SRR3502970 |
| SRP075075 | ncbi.nlm.nih.gov/sra | Hamilton MP | PAR-CLIP | human | DU145 | SRR3502975 |
| SRP075075 | ncbi.nlm.nih.gov/sra | Hamilton MP | PAR-CLIP | human | LAPC4 | SRR3502955, SRR3502965 |
| SRP033584 | ncbi.nlm.nih.gov/sra | Farazi TA | PAR-CLIP | human | MCF7 | SRR1045082 |
| GSE59944 | ncbi.nlm.nih.gov/geo | Whisnant AW | PAR-CLIP | human | TZMBL | GSM1462573, GSM1462574 |
| ERP002007 | ncbi.nlm.nih.gov/sra | Krell J | PAR-CLIP | human | HCT116 | ERR202916, ERR202920, ERR202924, ERR202928, ERR202932, ERR202936, ERR202940, ERR202944, ERR202948, ERR202917, ERR202921, ERR202925, ERR202929, ERR202933, ERR202937, ERR202941, ERR202945, ERR202949, ERR202914, ERR202918, ERR202922, ERR202926, ERR202930, ERR202934, ERR202938, ERR202942, ERR202946, ERR202915, ERR202919, ERR202923, ERR202927, ERR202931, ERR202935, ERR202939, ERR202943, ERR202947 |
| GSE98670 | ncbi.nlm.nih.gov/geo | Benway CJ | PAR-CLIP | human | HK2 | GSM2609180, GSM2609181, GSM2609182, GSM2609183 |
| GSE129076 | ncbi.nlm.nih.gov/geo | Muys BR | PAR-CLIP | human | A2780 | GSM3693008, GSM3693009, GSM3693010, GSM3693011 |
| GSE78059 | ncbi.nlm.nih.gov/geo | Gillen AE | HITS-CLIP | human | MCF7 | GSM2065789, GSM2065790,  GSM2065791,GSM2065792,  GSM2065793, GSM2065794 |
| GSE59944 | ncbi.nlm.nih.gov/geo | Whisnant AW | PAR-CLIP | human | C8166 | GSM1462572 |
| SRP009082 | ncbi.nlm.nih.gov/sra | Lipchina I | PAR-CLIP | human | ESC | SRR359787 |

**
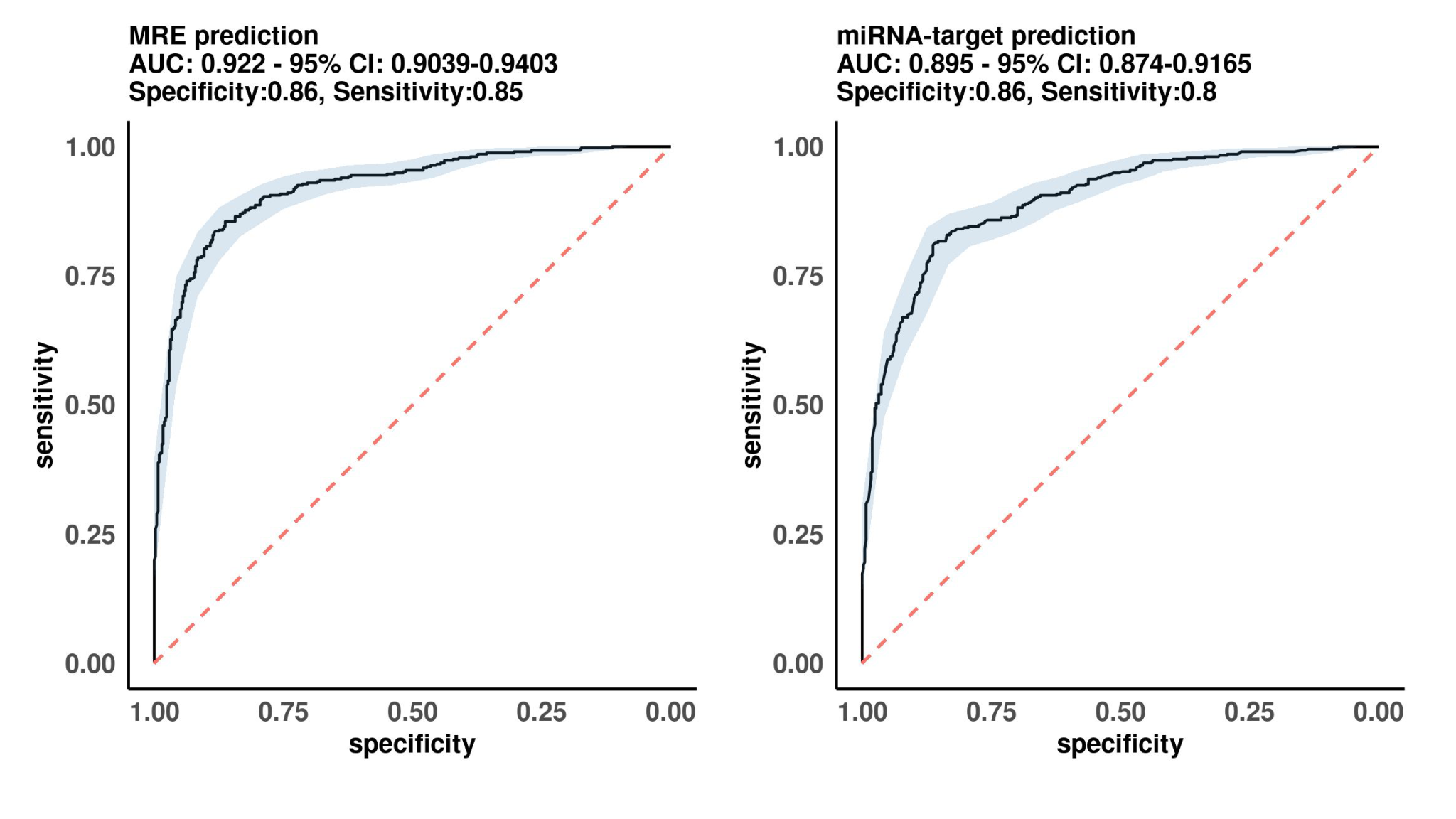
**

**Supplementary Figure 1: Evaluation of microT-CNN-detected miRNA targets.** Receiver Operator Characteristic (ROC) curves showing the ability of microT-CNN to accurately detect (left) Mirna Recognition Elements (MREs) and (right) miRNA-gene interactions. The model was evaluated in 830 MREs (415 positive miRNA-chimeric fragments and 415 negative miRNA binding sites), corresponding to 131 miRNAs and 727 unique genes. The Area Under the Curve (AUC) scores are shown for both predictions, along with the respective 95% confidence intervals (CIs), computed using 2000 stratified bootstrap replicates. Specificity and sensitivity values are displayed at the top of each AUC plot. Specificity and sensitivity were calculated by applying optimal thresholds to the microT-CNN scores based on the Youden index.


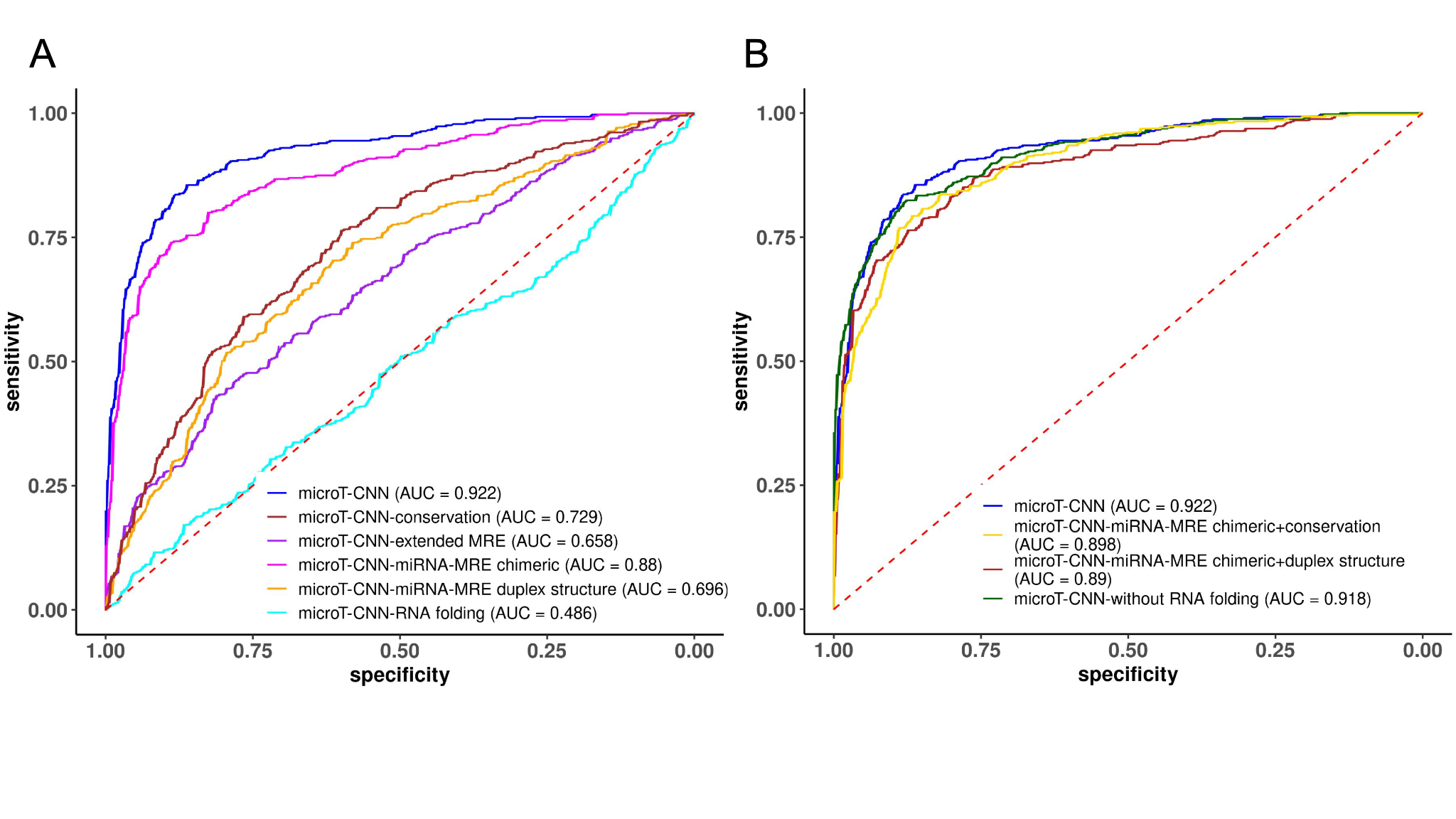


**Supplementary Figure 2: Evaluation of microT-CNN architecture following ablation experiments:** Receiver Operating Characteristic (ROC) curves illustrating the ability of distinct microT-CNN models to detect miRNA Recognition Elements (MREs). The models were evaluated using 830 MREs (415 positive miRNA-chimeric fragments and 415 negative miRNA binding sites), corresponding to 476 3' UTR regions, 354 CDS regions, 131 miRNAs, and 727 unique genes. (A) ROC curves comparing the performance of microT-CNN with models trained on the individual ‘expert’ branches integrated into microT-CNN. (B) ROC curves comparing microT-CNN with models trained on different combinations of the ‘expert’ branches integrated into microT-CNN. Distinct colors represent the different models, with Area Under the Curve (AUC) scores shown for each model.


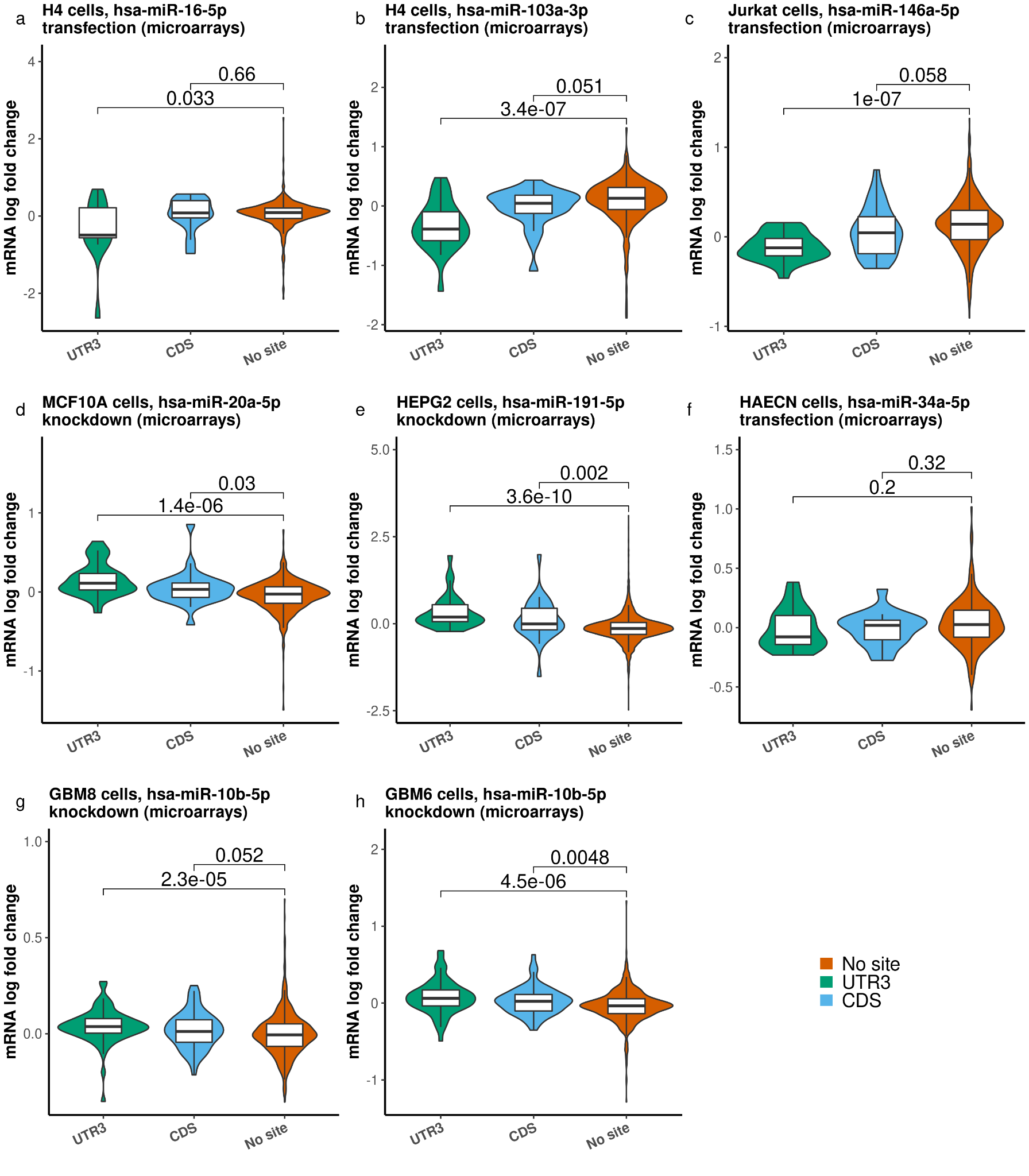


**Supplementary Figure 3: Functional efficacy of microT-CNN-detected miRNA targets residing on 3’ UTR or CDS regions.** The functional efficacy of the predicted targets was examined in 8 public gene expression profiling datasets following miRNA transfection or knockdown, corresponding to 7 cell types. The response of the top 100 microT-CNN-scored targeted mRNAs was evaluated (A-H). Boxplots of mRNA fold changes in a log2 scale for targets comprising at least one 3’ UTR MiRNA Recognition Element (MRE) or supported only by CDS MREs were compared to those that lack any site of the considered miRNAs. P-values for the differences in expression changes of the identified targets on 3’ UTR and CDS regions compared to transcripts lacking any predicted miRNA binding site are portrayed (two-tailed Wilcoxon rank-sum test).


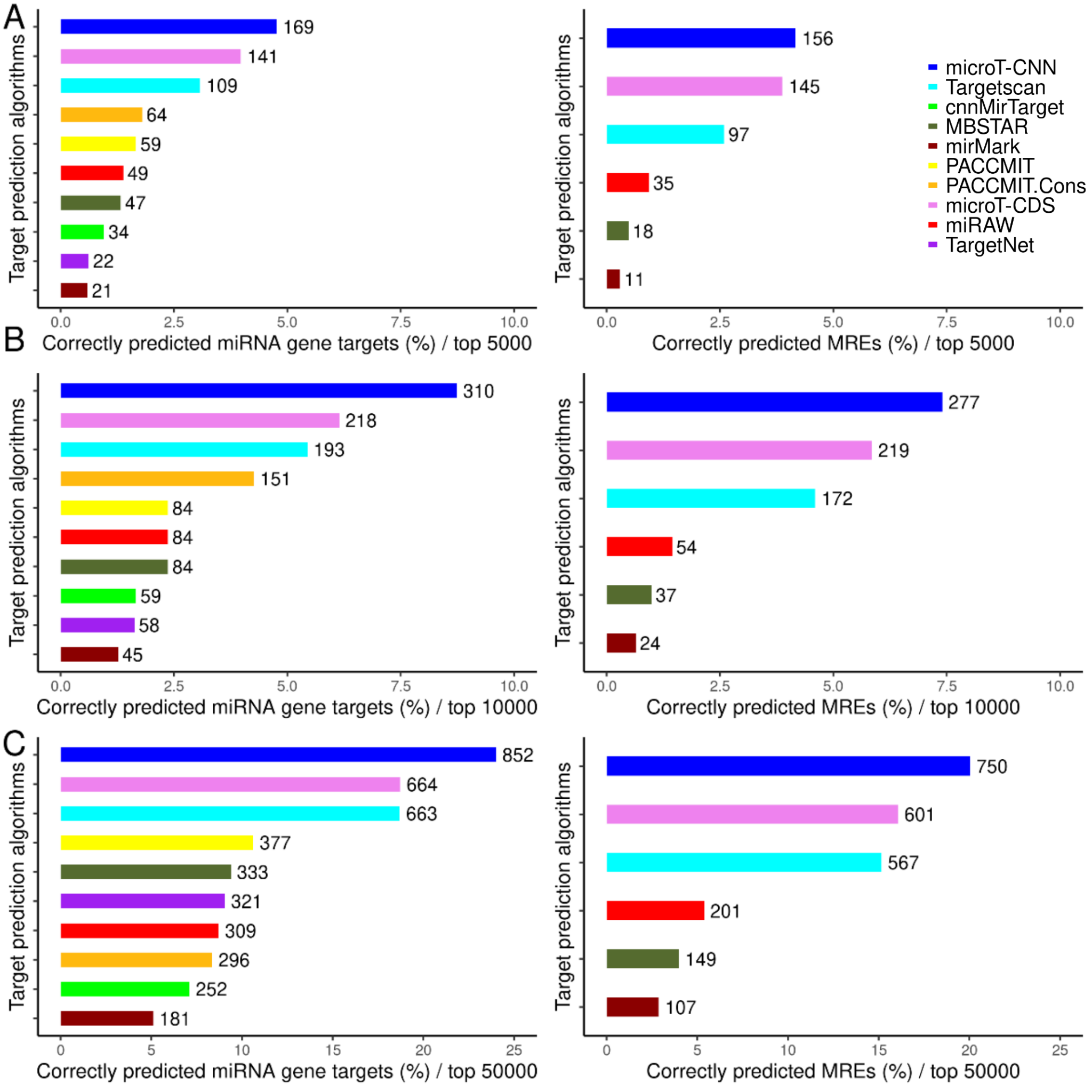


**Supplementary Figure 4:** Evaluation of microT-CNN performance against state-of-the-art implementations considering different cutoff thresholds regarding the top-scored miRNA targets provided by each model. The utilized validation set comprises 3,743 unique MiRNA Recognition Elements (MREs), corresponding to 3,548 miRNA-target interactions derived from 153 miRNAs supported by chimeric miRNA-gene fragments. (Left) The amount of correctly predicted miRNA gene targets and (Right) the correctly predicted MREs are portrayed *versus* the (A) 5,000, (B) 10,000, and (C) 50,000 top-scored miRNA targets provided by each model. The actual number of correctly predicted miRNA targets and MREs per model are portrayed in each barplot, respectively.


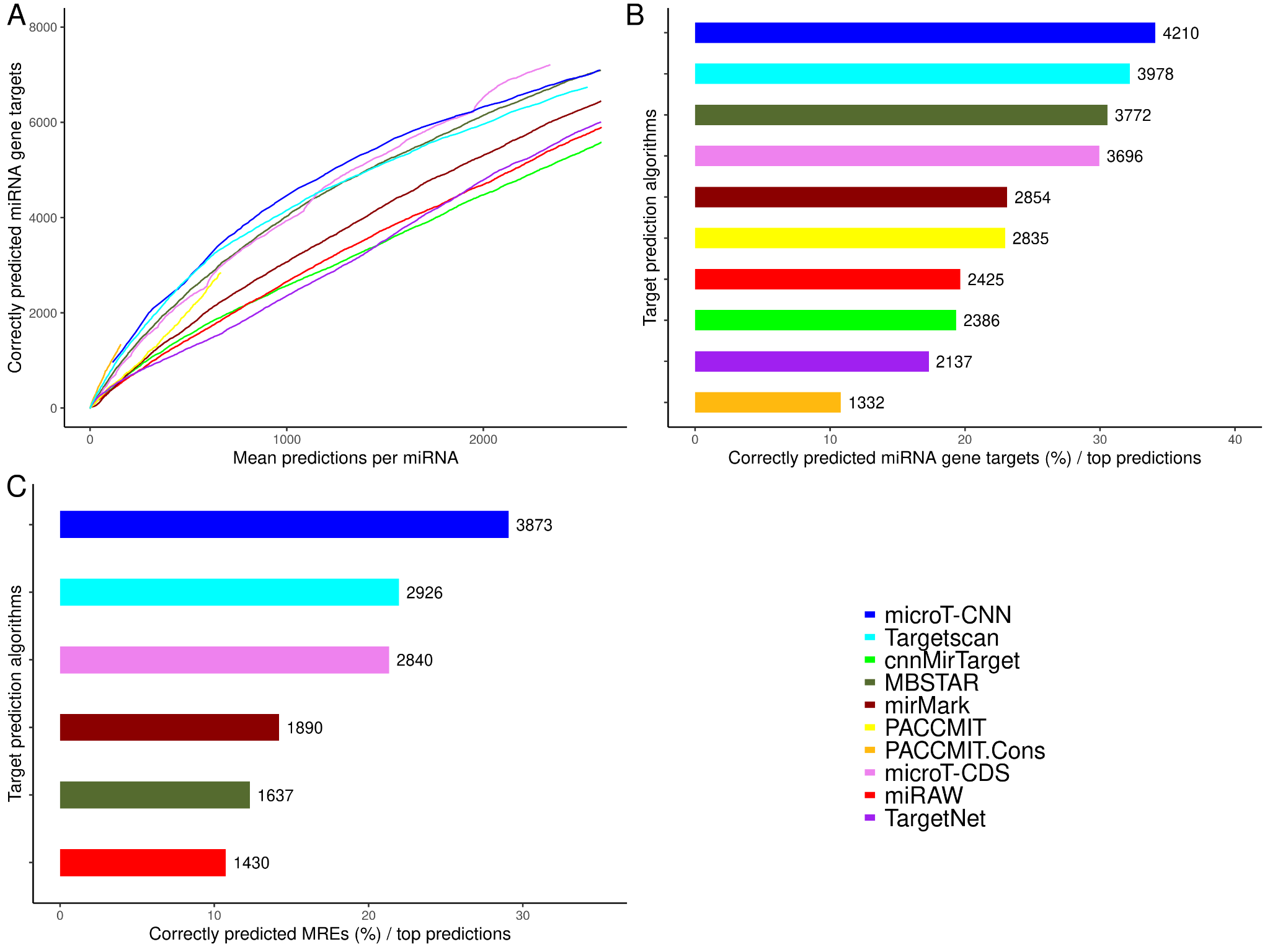


**Supplementary Figure 5:** microT-CNN juxtaposed against microT-CDS, Targetscan v7, miRAW, mirMark, PACCMIT-CDS, PACCMIT-CDS accounting for conservation, MBSTAR, and cnnMirTarget. The validation set consists of 13,357 AGO-binding regions, corresponding to 110 miRNAs, providing 12,343 unique miRNA-target interactions derived from 2 distinct cell types (C8166, hESC). (A) The number of correctly predicted miRNA gene targets *versus* the mean predictions per miRNA is portrayed. (B) The percentage of the correctly predicted miRNA-gene interactions *versus* the top-scored 100,000 predictions per model is displayed. The actual number of correctly predicted miRNA-gene interactions is displayed per model. (C) The percentage of the correctly predicted MREs at the top-scored 100,000 miRNA-gene predictions per model is portrayed. The actual number of correctly predicted MREs is displayed per model.
